# A Hybrid Model for Vehicle Acceleration Prediction

**DOI:** 10.3390/s23167253

**Published:** 2023-08-18

**Authors:** Haoxuan Luo, Xiao Hu, Linyu Huang

**Affiliations:** 1 College of Electronics and Information Engineering, Sichuan University, Chengdu 610065, China; hxluo@stu.scu.edu.cn; 2 College of Information Science and Technology, Northeast Normal University, Changchun 130117, China; hux192@nenu.edu.cn

**Keywords:** acceleration prediction, clustering, driving behavior, neural network, data-driven method, Gaussian mixture models

## Abstract

Accurate prediction of vehicle acceleration has significant practical applications. Deep learning, as one of the methods for acceleration prediction, has shown promising applications in acceleration prediction. However, due to the influence of multiple factors on acceleration, a single data model may not be suitable for various driving scenarios. Therefore, this paper proposes a hybrid approach for vehicle acceleration prediction by combining clustering and deep learning techniques. Based on historical data of vehicle speed, acceleration, and distance to the preceding vehicle, the proposed method first clusters the acceleration patterns of vehicles. Subsequently, different prediction models and parameters are applied to each cluster, aiming to improve the prediction accuracy. By considering the unique characteristics of each cluster, the proposed method can effectively capture the diverse acceleration patterns. Experimental results demonstrate the superiority of the proposed approach in terms of prediction accuracy compared to benchmarks. This paper contributes to the advancement of sensor data processing and artificial intelligence techniques in the field of vehicle acceleration prediction. The proposed hybrid method has the potential to enhance the accuracy and reliability of acceleration prediction, enabling applications in various domains, such as autonomous driving, traffic management, and vehicle control.

## 1. Introduction

With the rapid development of sensor technology, how to use the data obtained by sensors is a crucial issue, and data prediction is an important part of it. Take Hybrid Electric Vehicles for example, the Energy Management Strategy determines power allocation among multiple energy sources and is crucial for lowering fuel consumption and emissions, in which a data-based model is often used [1,2]. The Internet of Vehicles is also part of the Internet of Things, and Advanced Driver Assistance Systems, Automated Driving Functions, and Intelligent Driver Assistance are important techniques [3]. Vehicle acceleration is one of the most essential input characteristics for the subsystems above [4,5]. Consequently, the accuracy of the acceleration state, both in the detection of the present state and the prediction of the future state, has a direct impact on system performance [6]. Current state detection is straightforward and can be easily accomplished via sensors. However, the prediction of future states is much more challenging.

Commonly employed prediction approaches are typically separated into model-based methods and data-based methods [7,8]. The Markov Chain, Conditional Linear Gaussian (CLG), and Auto-Regressive Moving Average models are widely used model-based prediction techniques. The MC and CLG are stochastic prediction algorithms that can generate prediction error distribution characteristics in addition to forecasting the mean [9]. Some studies directly establish a linear model in a specific context. Ref. [10] developed a model for estimating the speed of vehicles as they turn on rural roads. Due to their limited complexity and inability to fully utilize the dataset’s information, model-based prediction approaches require far less training data than data-based methods.

Without fixed parameters or structures, data-based prediction systems usually depend on the characteristics of the data. For example, Ref. [11] developed a Convolutional Neural Network (CNN)-based modular structure With Deep Neural Network (DNN) models. Ref. [12] compared the outcome of speed prediction using Recurrent Neural Networks (RNN) and Long Short-Term Memory (LSTM) on the highway, showing that LSTM has better short-term speed prediction performance and higher accuracy than RNN. The LSTM Neighbors developed by [13] are based on the LSTM networks, which can use more car-following data. Furthermore, their outcome is significantly more optimal than the LSTM networks, which only utilize the information of the vehicle directly in front of the ego vehicle. Ref. [14] proposed a novel online vehicle velocity prediction strategy based on an online trained Radial Basis Function Neural Network (RBF-NN) with an adaptive structure, which not only improves the prediction accuracy of RBF-NN with an adaptive structure but also gives the prediction outcome in a shorter time. Ref. [15] used LSTM networks and Gated Recurrent Unit (GRU) networks to forecast the short-term traffic flow. Ref. [8] separated the drivers into two groups and subsequently predicted vehicle acceleration using LSTM and GRU networks, respectively. However, in general, the aforementioned methods require a considerable amount of time to train a model, and the resulting model cannot be applied to multiple driving scenarios, which adds complexity and instability to the prediction process. With the continuous development of clustering technology, more and more people study acceleration prediction by combining deep learning and clustering technology [16]. This research draws on the decision-making behavior of human drivers with motivation at the core and proposes a decision-making and planning method based on motivation and risk assessment. This can provide a clear pattern and structure for subsequent acceleration prediction work. However, although clustering technology can make the acceleration prediction model more accurate, the model used by the above method is relatively simple and does not take into account the possibility that different clustering data and different driving scenarios are suitable for different networks. Therefore, it may not have better prediction performance on some specific distributed data.

To address the aforementioned issues and improve prediction accuracy, this paper proposes a hybrid acceleration prediction model based on clustering and deep learning. Since different prediction networks based on deep learning work best with different data features, we first clustered historical driving data to group together data with similar acceleration patterns. Then, different prediction networks are used for different clusters of data. Unlike existing work that partitions data based on drivers, our work clusters data based on the characteristics of vehicle acceleration data itself [8]. Since the raw data used in this study contain ten different driving features, normalization is adopted to ensure that each feature has a similar impact on clustering. Additionally, the probability of each data segment belonging to the Gaussian mixed model cluster is calculated. The main contributions of this paper can be summarized as follows:

- Given the variety of acceleration patterns, this study proposes a method for predicting acceleration that uses a combination of clustering and deep learning to improve accuracy.
- By clustering driving data into groups based on their features and then using different prediction networks on each group, the experiment showed that different prediction networks work well with driving data that have different features.
- The probability of each data segment belonging to each Gaussian mixed model cluster is calculated, and then the data are taken as a new input to the neural networks.
- Extensive experiments were carried out using a public database, and the performance of the proposed method was compared to that of unclustered data and existing works. This showed that the proposed method is better at improving the accuracy of predicting acceleration.

The remainder of the paper is structured as follows: Section 2 introduces the basic idea and workflow of the proposed method. Section 3 describes the dataset and preprocessing procedures. Section 4 provides a detailed description of the data features, dimensionality reduction operations, and clustering methods. Section 5 outlines how deep learning networks are employed to predict acceleration. Section 6 provides a detailed description of the experimental setup and performance analysis comparisons. Finally, Section 7 presents the conclusion and discussion.

## 2. Methodology Description

**Figure 1:**
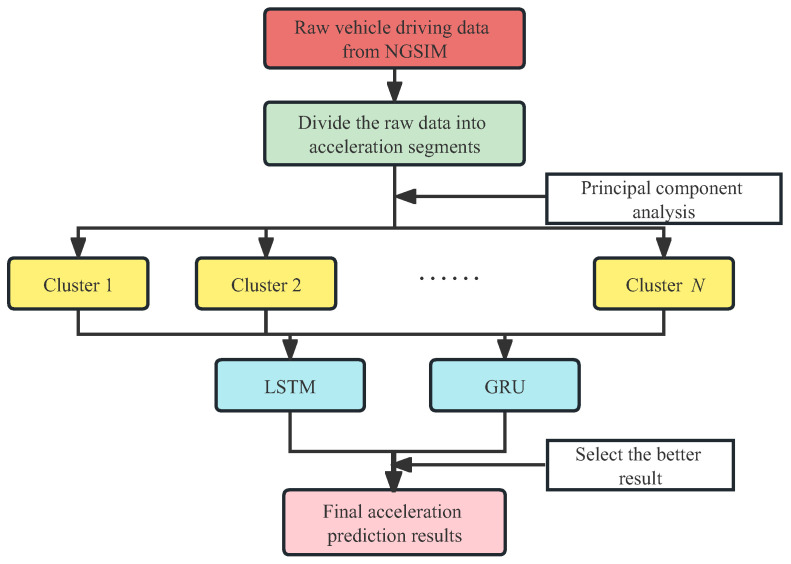
Flowchart of the proposed clustering-prediction hybrid model.

This paper proposes a hybrid method that combines clustering and deep learning to improve the accuracy of acceleration prediction. In this section, we will introduce the basic idea and general process of the method. Figure 1 shows the workflow of the method, which can be divided into three phases: data preparation, clustering, and prediction.

The main task of the data preparation phase is to divide the long-term driving data into short acceleration process segments. Each raw record in the database has a long driving process that may include more than one acceleration. In order to facilitate the analysis of each short acceleration process and the subsequent deep learning, the original data are segmented based on some criteria so that the time length of each segment can basically contain a short acceleration process. The specific segmentation criteria will be detailed in a later section.

The goal of the clustering phase is to classify the segmented data based on driving characteristics so that different classes have distinctly different feature values. Numerous factors affect each acceleration process [17]. Not only previous acceleration data but also current vehicle speed and the surrounding environment, such as distance from surrounding vehicles, have an impact. To improve the accuracy of prediction, this paper selects 10 out of the 16 features. It is unclear which of these numerous data features has a greater or more direct impact on acceleration. In order to improve the effectiveness and efficiency of clustering, all feature vectors are normalized.

There are numerous clustering algorithms with varying degrees of effectiveness, which can essentially be classified as supervised learning and unsupervised learning. Since supervised learning requires prior explicit knowledge of the category information of each classification and the premise that all items to be classified belong to a category, it is frequently impossible or prohibitively expensive to satisfy the aforementioned conditions when working with large amounts of data [18].

When working with large datasets and without a clear classification criterion, unsupervised learning, namely clustering, is frequently utilized for classification [19]. In this paper, the Gaussian Mixture Clustering (GMM) method is chosen to cluster the segmented data based on the three selected principal components. An important parameter during the clustering process is the number of categories to be clustered. This paper uses the AIC and BIC criteria to determine this parameter. Additionally, regularization parameters are used during the clustering process to prevent model overfitting. The details will be introduced in Section 4.

The last phase is to use deep learning to train and predict using each cluster’s acceleration data segments. Since each cluster of driving data has different features, the performance of different prediction networks for a given cluster may be very different. In this paper, we use two candidate networks, LSTM and GRU, to train and predict separately for each cluster’s data and select the one with better prediction performance for each cluster. To evaluate the prediction performance, the Root Mean Square Error (RMSE) and Mean Absolute Error (MAE) are chosen as evaluation metrics.

To verify the clustering effect, we directly applied LSTM and GRU to the entire dataset without clustering and compared the results with those after clustering with the proposed method. To verify that different prediction models are suitable for different clusters, we applied LSTM and GRU separately to each cluster and compared and analyzed the results. To fully evaluate the performance of our proposed method, we also compared the prediction accuracy of some typical algorithms.

Details will be provided in the following sections.

## 3. Dataset and Preprocessing

The dataset we used is based on the Next Generation Simulation (NGSIM) database [20]. The data were collected from four distinct locations: the southbound US 101 in California, the Lankershim Boulevard map in Los Angeles, the eastbound I-80 in Emeryville, California, and Peachtree Street in Atlanta, Georgia. The NGSIM dataset comprises structured road intersections, high-speed upstream and downstream lanes, and high-speed sections. The NGSIM dataset has high data accuracy and diverse data types that can describe vehicle motion processes in detail. The collected data are sampled at a rate of 10 Hz, and the collected data include vehicle location, vehicle number, etc. The dataset is large enough to encompass various scenes, with each scene containing sufficient data.

Due to the imperfection of the original dataset, which includes errors, redundant data, and missing data, the errors will be amplified when using neural networks to predict speed and acceleration, thus affecting the accuracy of the predictions. Therefore, before proceeding with further processing of the original dataset, a dataset-cleaning step is necessary. Redundant and erroneous data are removed, and missing frames with fewer than 10 occurrences are filled using corrected Akima piecewise cubic Hermite interpolation [21].

It should be noted that each frame represents 0.1 s because the data are sampled at a rate of 10 Hz. The preprocessed dataset consists of *N* frames of data, and each frame includes the following information: data index *i*, vehicle velocity $v_{i}$, vehicle acceleration $a_{i}$, and distance to the preceding vehicle $s_{i}$. The entire dataset is divided into *n* sample segments, some criteria for segmenting data are demonstrated as follows:

(1) To ensure that the data used for subsequent clustering and prediction includes complete acceleration or deceleration processes, data segments with a travel time of less than 60 s are excluded.

(2) We consider that the influence of the preceding vehicle on driving behavior is not significant when the distance exceeds 80 m. Therefore, in the data records, distances exceeding 80 m from the preceding vehicle are treated as 80 m.

Based on the aforementioned criteria, we set that each segment contains 200 frames of data. In subsequent processing, we treat each segment as a unit when clustering but extract features using frames in each segment in Section 4, and predict a single frame in each segment in Section 5 and Section 6.

## 4. Feature Extraction and Clustering

### 4.1. Characteristic Parameters Extraction

**Figure 2:**
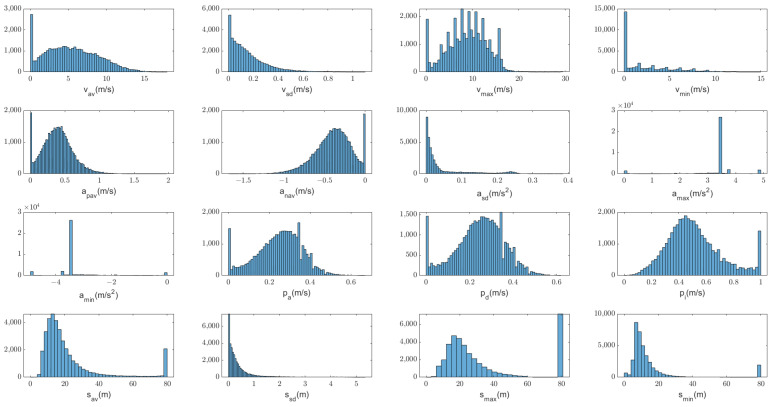
Statistical distributions of characteristics.

Although we attempt to estimate acceleration, it is insufficient to rely solely on acceleration data. Therefore, we first calculate the commonly used feature parameters of each data segment, i.e., average velocity $v_{av}$, velocity standard deviation $v_{sd}$, maximum velocity $v_{max}$, minimum velocity $v_{min}$, average positive acceleration $a_{pav}$, average negative acceleration $a_{nav}$, acceleration standard deviation $a_{sd}$, maximum acceleration $a_{max}$, minimum acceleration $a_{min}$, acceleration time ratio $p_{a}$, deceleration time ratio $p_{d}$, idle time ratio $p_{i}$, average vehicle spacing $s_{av}$, vehicle spacing standard deviation of $s_{sd}$, maximum vehicle spacing $s_{max}$, and minimum vehicle spacing $s_{min}$. Please refer to the Appendix A for detailed definitions of the above parameters. The statistical histograms of the features are shown in Figure 2. Among them, several features with relatively scattered distributions are finally selected as the primary parameters reflecting driving behavior patterns. These parameters include $v_{av}$, $v_{sd}$, $v_{max}$, $a_{pav}$, $a_{nav}$, $p_{a}$, $p_{d}$, $p_{i}$, $s_{av}$, and $s_{max}$.

After feature extraction, a dataset consisting of feature parameters is formed, with a total of *n* frames. Each frame contains the feature parameters extracted from the corresponding data segment.

### 4.2. Normalization

The features used include vehicle speed, acceleration, and distance to the preceding vehicle, with significant differences in the numerical values of different features. In order to ensure that each feature has a comparable impact on clustering, it is necessary to normalize the features. The normalization formula used is as follows:

(1) $$x_{r}=q+\frac{x_{i}-x_{min}}{x_{max}-x_{min}}\times \left(p-q\right),$$

In the normalization process of the extracted features for vehicle acceleration, q=−0.5 and p=0.5 are used. In the normalization process of the extracted features for vehicle speed and distance to the preceding vehicle, q=0 and p=1 are used.

The dataset consisting of feature parameters is now transformed into a dataset consisting of the normalized components, denoted as $X=\{x_{1},x_{2},\ldots ,x_{n}\}$, where $x_{j}=(v_{avj},\;v_{sdj},\;v_{maxj},\;$$a_{pavj},\;a_{navj},p_{aj},\;p_{dj},\;p_{ij},\;s_{avj},\;s_{maxj}).$

### 4.3. Clustering Method

There are various clustering methods available for data analysis. GMM is not only more flexible in splitting the shape of clusters but also takes into consideration the data variance and is unbiased [22]. Considering the large amount of data and heterogeneous features, GMM clustering is chosen as the clustering method for the dataset. GMM is a type of unsupervised learning algorithm that divides a dataset into multiple Gaussian distribution mixtures. Each mixture can be represented as a Gaussian distribution with its own mean and covariance matrix. During the clustering process, the model determines the number of clusters and the center positions and shapes of each cluster by iteratively solving for the maximum likelihood of the likelihood function.

Because Gaussian Mixture Clustering requires solving for the maximum likelihood of the likelihood function, it can be implemented using the Expectation-Maximization (EM) algorithm.

First, we set the parameters of the Gaussian Mixture Model, including the number of mixtures *k*, the mean $\underset{-}{≺}=\{\mu_{1},\mu_{2},\ldots ,\mu_{k}\}$ and covariance matrix $\Sigma =\{\Sigma_{1},\Sigma_{2},\ldots ,\Sigma_{k}\}$ of each mixture, and the mixing weights $\underset{≉}{≻}=\{\pi_{1},\pi_{2}\ldots \pi_{k}\}$.

Then, the Expectation (E) Step: calculate the probability of each data point belonging to each mixture and calculate the posterior probability of each data point belonging to each cluster using Bayes’ theorem. As we have *n* data points and *k* clusters, the probability that the *i*-th data point x(i) belongs to the *j*-th cluster is:

(2) $$P\left(z_{j}|x_{i}\right)=\frac{P\left(x_{i}|z_{j}\right)P\left(z_{j}\right)}{\sum_{l=1}^{k}P\left(x_{i}|z_{l}\right)P\left(z_{l}\right)}$$

where $z_{j}$ is the *j*-th Gaussian mixture component or cluster, $x_{i}=\left(h_{1i},h_{2i}\right)$ is the *i*-th data point, $P(z_{j})$ is the prior probability of choosing the *j*-th mixture component, and $P(x_{i}|z_{j})$ is the probability density function of the *i*-th data point on the Gaussian distribution given the *j*-th mixture component, which is:

(3) $$P\left(x_{i}|z_{j}\right)=\frac{exp(-\frac{1}{2}{\left(x_{i}-\mu_{j}\right)}^{T}\Sigma_{j}^{-1}\left(x_{i}-\mu_{j}\right))}{{\left(2\pi \right)}^{\frac{k}{2}}{\left|\Sigma_{j}\right|}^{\frac{1}{2}}}$$

Then, the Maximization (M) Step: Based on the posterior probability obtained in the E step, update the mean, covariance matrix, and mixing weight of each cluster. Specifically, for the parameters of the *j*-th cluster, we have:

(4) $$\mu_{j}=\frac{\sum_{i=1}^{n}P\left(z_{j}|x_{i}\right)x_{i}}{\sum_{i=1}^{n}P\left(z_{j}|x_{i}\right)}$$

(5) $$\Sigma_{j}=\frac{\sum_{i=1}^{n}P\left(z_{j}|x_{i}\right)\left(x_{i}-\mu_{j}\right){\left(x_{i}-\mu_{j}\right)}^{T}}{\sum_{i=1}^{n}P\left(z_{j}|x_{i}\right)}$$

(6) $$\pi_{j}=\frac{1}{n}\sum_{i=1}^{n}P\left(z_{j}|x_{i}\right)$$

Utilize the *E* and *M* steps to iteratively update until convergence, which was determined by the change in the model likelihood function.

To address the issue of correlation between features, regularization is used to help mitigate multicollinearity and thereby promote the selection of independent and informative features [23]. The regularization parameter is set to 0.01.

### 4.4. Clustering Result and Analysis

Although GMM is not particularly sensitive to the choice of the number of clusters *k*, selecting the appropriate number of clusters is crucial for effectively classifying vehicle driving situations and predicting vehicle acceleration in subsequent steps. Therefore, the model selection tools, Akaike Information Criterion (AIC) and Bayesian Information Criterion (BIC) are used to assist in determining the optimal number of clusters [24]. They are likelihood-based model fit measures that incorporate a complexity penalty, specifically the number of parameters.

The number of estimated parameters is the AIC, while the BIC indicates the quantity of observations and calculated parameters. They are defined as follows:

(7) AIC=2×h−2×logL

(8) BIC=h×log(n)−2×logL

where *n* is the number of driving data segments and *h* is the number of parameters, which means the principal components, i.e., 2 in this study. logL is the probability density function value of the data sample given a particular model parameter, which can be represented as:

(9) $$L=\sum_{i=1}^{n}\sum_{j=1}^{k}\pi_{j}\times G\left(x_{i}|\mu_{j},\Sigma_{j}\right)$$

where $G(x_{i}|\mu_{j},\Sigma_{j})$ refers to the density function of multivariate Gaussian distribution, representing the distance between the sample point $x_{i}$ and the *j*-th Gaussian distribution.

When comparing multiple models, the one with a lower value of AIC and BIC is better. The measured AIC and BIC of these five clusters for our dataset are shown in Table 1.

**Figure 3:**
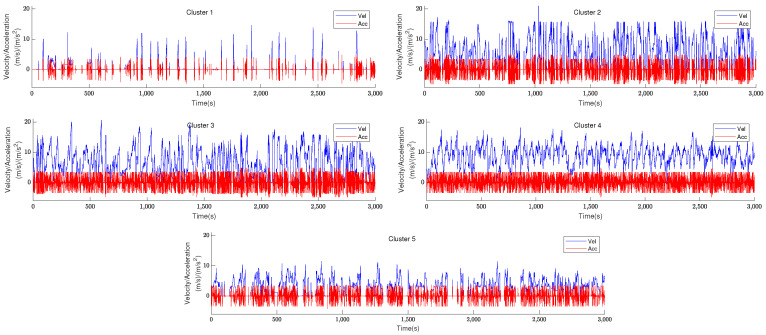
Part of the vehicle velocity and acceleration from different clusters.

It can be observed that the AIC and BIC values tend to decrease before the number of clusters approaches 5, and that their values increase when the number of clusters exceeds 5. Therefore, the number of clusters is fixed to 5. The corresponding velocity and acceleration image after clustering is shown in Figure 3.

Figure 3 roughly shows that each of the five clusters has its own unique features, with the longest idle time in Cluster 1, the highest average speed and relatively erratic acceleration in Cluster 2, the highest absolute value of average acceleration in Cluster 3, generally smooth velocity and acceleration in Cluster 4, and the lowest speed in Cluster 5. Next, we make predictions for each of the five clusters based on their individual characteristics.

In real applications, data can be collected online through a shared system. When each vehicle collects a sufficient amount of data, the vehicles can use their own driving data to build models as well. At first, the real-time data are first divided into segments of 20 s each. Missing frames in segments with few missing values are filled using corrected Akima piecewise cubic Hermite interpolation. Additionally, data points with a distance greater than 80 m to the preceding vehicle are replaced with 80 m. Then, the feature values are computed for each data segment, and all the features are normalized using the same method mentioned before. Since the weights $\approx =\{\pi_{1},\pi_{2},\pi_{3},\pi_{4},\pi_{5}\}$ of each Gaussian distribution have been obtained, the probability of an external data point $x_{i}$ belonging to the *k*-th Gaussian distribution is computed using. Finally, as each cluster’s mean $\underset{-}{≺}=\{\mu_{1},\mu_{2},\mu_{3},\mu_{4},\mu_{5}\}$ and covariance matrix $\Sigma =\{\Sigma_{1},\Sigma_{2},\Sigma_{3},\Sigma_{4},\Sigma_{k}\}$ are known, the probability of each segment belonging to each cluster can be calculated:

(10) $$P_{k}\left(x_{i}\right)=\pi_{k}\times G\left(x_{i}|\mu_{i},\Sigma_{k}^{2}\right),$$

where *G* represents the Gaussian distribution. The Gaussian distribution with the highest weight for $x_{i}$ corresponds to the class to which it belongs.

## 5. Prediction Based on Deep Learning

This section will describe how to anticipate acceleration for clustered data based on machine learning. Due to the continuous nature of driving, the deep learning network model based on vehicle data for this project must be capable of fitting time series data appropriately and effectively. Since each cluster of data has its unique features, no network can perform optimally on all clusters in general. Thus, the methodology of evaluating the prediction performance of various neural networks on distinct clustering data is adopted, and then the most suitable model is selected for each cluster based on its performance. In this study, the performance of the four networks in the clustering-prediction hybrid model is compared, and the comparison procedure is detailed below.

Under the framework of acceleration prediction tasks, not all networks can achieve satisfactory performance. Although one-dimensional CNN networks have been used in some papers for acceleration prediction, CNN networks are not fully suitable for the sequence here. CNN is primarily designed for image processing tasks, focusing on capturing spatial relationships. In the case of vehicle acceleration prediction, which involves time series data, CNN cannot directly model sequences, as it lacks explicit mechanisms to handle temporal dependencies and sequence evolution [25]. CNN’s pooling layers and downsampling operations can reduce the size of feature maps, which helps reduce computational costs. However, this can also lead to information loss. In vehicle acceleration prediction, each timestep’s acceleration value carries important contextual information, and CNN may not retain the complete time series information. Although RNN networks are commonly used for handling time series data, they also have some limitations. Standard RNNs are prone to the problem of vanishing or exploding gradients during the training process. This is caused by the exponential decay or growth of gradients over time during backpropagation. This issue makes it challenging for RNNs to capture long-term dependencies, which are crucial for vehicle acceleration prediction [26]. All of these factors may contribute to poor network performance during later vehicle acceleration prediction [27].

In contrast, LSTM and GRU networks offer distinct advantages in vehicle acceleration prediction. Due to their gated mechanisms, LSTMs and GRUs can effectively capture long-term dependencies in driving sequences, which is crucial for predicting acceleration patterns that may span across various time intervals. Their memory cells and gating mechanisms allow them to retain relevant contextual information and process sequential data more effectively than traditional RNNs. By using an LSTM-GRU hybrid network, we can potentially leverage the strengths of both architectures, benefiting from the memory and context preservation of LSTMs while enjoying the simplified structure of GRUs [28]. This hybrid approach may improve the accuracy and performance of acceleration prediction models, enhancing their applicability in real-world scenarios. In subsequent experiments and model selection, LSTM and GRU models demonstrated superior performance.

### 5.1. Network Structure

In the previous section, we compared the differences between various networks and explained the reasons for choosing the LSTM and GRU networks over other networks. In the following, we will provide a detailed description of the specific structure of the LSTM and GRU networks.

We define the driving process as the process of modifying the vehicle indicators over time. In this experiment, we use pre-clustering and post-clustering data with varying lengths of driving data segments as input to the hybrid network to train and compare the performance of several acceleration prediction networks. Experiments demonstrate that the accuracy of predicting acceleration is greatly enhanced after clustering compared to before clustering.

Recall that we divide the corresponding sample data segments into multiple classes based on the clustering results. Each frame of data, represented as $y_{i}=\left(v_{i},a_{i},s_{i}\right)$, captures the variations in vehicle motion over 200 frames. We define the driving process as the modification of vehicle indicators over time. In this experiment, we use pre-clustered and post-clustered data with varying lengths of driving data segments as input to the hybrid network. The objective is to train and compare the performance of several acceleration prediction networks.

In this experiment, each data segment represents the motion process of a car within 20 s. Therefore, we used a hybrid prediction network to extract and shape features from the first 150 frames of the data segment to learn the car motion process. Then, the network generates vectors representing predicted future acceleration values based on the motion process. In order to verify the performance superiority of this network in short-term and long-term prediction, we used data from the last 20 frames and 50 frames of the data segment as an evaluation of actual results, representing the performance of the network in two scenarios: short-term prediction (2 s) and long-term prediction (5 s), respectively.

Since clustered data with different data characteristics may perform differently under various networks, we compare the performance of the LSTM and GRU networks for each clustered data type to choose the network that performs better. By utilizing LSTM and GRU networks in a hybrid architecture, one can leverage their strengths in handling long-term dependencies and preserving contextual information, thereby improving the accuracy and performance of vehicle acceleration prediction models. Hence, we select the LSTM-GRU hybrid network as the primary network for this experiment to achieve better performance. The following sections will provide a detailed review of the LSTM network and the GRU network.

### 5.2. Realization of Prediction Based on LSTM Networks

Instead of focusing on the performance of the network models themselves, the objective of this study is to compare the performance of prediction models based on pre-clustering and post-clustering datasets. Therefore, LSTM networks and fully connected layers are introduced in the clustering-prediction mixed model as a deep learning model for acceleration prediction.

The LSTM network, a special variant of RNN network, is often used to process sequence data and solve long-term dependency problems. It has been used in many experiments for acceleration prediction and has shown excellent performance. The core idea of LSTM is to introduce memory cell and gate structures to better capture and process long-term dependencies in sequences. Compared to traditional RNN, LSTM has strong memory ability and can better preserve and utilize past information.

**Figure 4:**
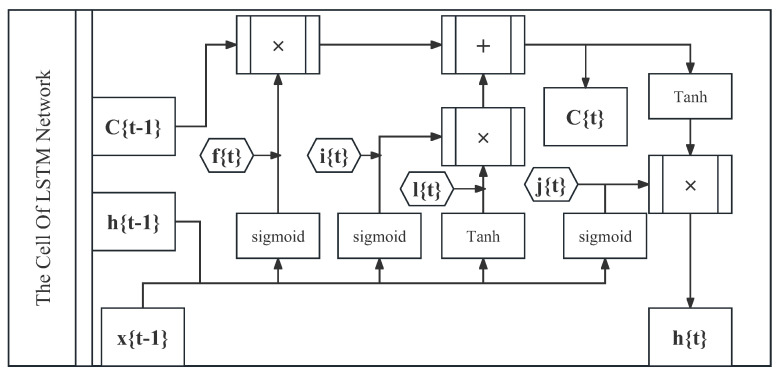
Structure of LSTM.

The LSTM network framework used for acceleration prediction, also known as gate structures [27], mainly includes Input Gate, Forget Gate, Output Gate, and Memory Cell. Each gate has corresponding weights and biases to control the flow of information. With these gating structures, LSTM networks are able to flexibly determine the input, output, and state updates at each time step, thus better handling long-term dependencies. For the driving data in this study, the LSTM network can retain and transmit the important information of the vehicle driving state in the earlier time in the data segment via unique gate structures, so that the state information in the earlier time can still contribute to the acceleration prediction. This makes the LSTM network obtain a good effect in processing the driving data. The use of an LSTM network can alleviate the problem of gradient vanishing to a certain extent, and can also improve the accuracy of acceleration prediction. The LSTM network structure is shown in Figure 4. As mentioned earlier, we use the first 150 frames of the data segments as input to train the LSTM network.

In practice, the overall trend of acceleration prediction network performance shows that as the number of training epochs and neurons increases, the speed of network performance optimization slows down and even declines. The reason is that the data feature dimension used for acceleration prediction is smaller, and when the number of neurons is much higher than the feature dimension and the number of training epochs is too large, the network is easy to overfit, resulting in a poor acceleration prediction effect. The parameters used to construct the LSTM network are displayed in Table 2. The LSTM network used in this experiment is constructed in a PyTorch-based deep learning environment running on Windows.

### 5.3. Realization of Prediction Based on GRU Networks

The experimental results show that only introducing the LSTM network into the clustering-prediction mixed model for acceleration prediction has great limitations. The performance of an acceleration prediction model needs to be judged not only by the prediction accuracy of the model, but also by the time required for training the model and making predictions. Shorter time requirements usually mean better adaptability, where the LSTM network tends to fit slowly in tests predicting acceleration. Therefore, the GRU network, which in general takes less time than an LSTM network, is also selected as a candidate prediction network for the clustering-prediction mixed model.

The GRU network is a uniquely enhanced and accelerated LSTM network. Similar to LSTM, GRU is also a chain model composed of repeated gated structural unit modules. However, GRU combines the forget gate and input gate of the LSTM gate structure, enabling the network to have fewer parameters. Therefore, compared to the LSTM network, GRU has unique improvements in handling driving data. The improvement of the network is reflected in the shorter fitting time of the GRU network for subsequent acceleration prediction on driving data, providing it with a distinct advantage when dealing with large batches of training data [29].

The addition of the GRU network provides a novel contrast to this experiment, as the GRU network tends to have a faster fit in tests predicting acceleration using neural networks trained on historical traffic data. In trials utilizing a neural network trained on historical traffic data, the GRU network tends to fit faster. It produces diverse outcomes for various data clusters and LSTM, and it performs better when clustering data with a specified distribution. For clustering data with different distributions, one of the LSTM network or the GRU network can be selected to achieve a more optimized acceleration prediction performance, which enables the clustering-prediction mixed model to have better versatility.

**Figure 5:**
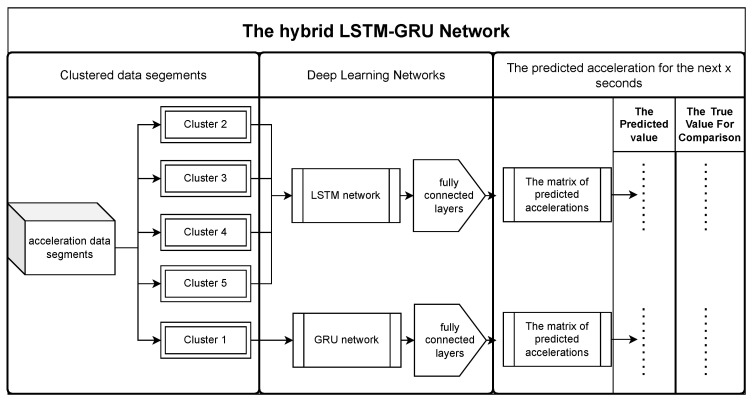
Structure of the network for acceleration prediction based on different clustered vehicle driving data.

The platform and related parameters used to build the GRU network are the same as those used to build the LSTM network. We train the GRU network using the same fixed-length data segments of 150 frames as with the LSTM network, so as to control variables and realize the comparison between different models. The network structure is shown in Figure 5.

## 6. Validation and Discussion

### 6.1. Preparation for Experiments

The first step in prediction is to select an appropriate input data length for the neural network as well as the time length of the data to be predicted. Too long or too short time lengths can have an impact on the network’s prediction performance and practical value. As a result, taking into account the demand for prediction duration in practical application scenarios, we use 10 s of historical data to predict 2-s and 5-s accelerations, respectively, to evaluate the network model’s performance in predicting short and longer periods.

The dataset used in this experiment consists of a total of 37,526 acceleration segments, which are obtained based on the NGSIM database. We processed the dataset by randomly dividing 70% of it as the training set and the remaining 30% as the test set. For each cluster’s data, different networks are trained and tested independently.

Driver intentions and behaviors can differ greatly depending on the driving environment and road conditions, so the data characteristics of different clusters may also differ. Deep learning neural networks built on different clusters of data will produce different prediction results. In order to explain and compare the quality of prediction results, RMSE and MAE are introduced as evaluation metrics, which are defined as follows:

(11) $$\mathrm{RMSE}=\sqrt{\frac{1}{m}\sum_{i=1}^{m}{\left(y_{i}-{\hat{y}}_{i}\right)}^{2}}$$

(12) $$\mathrm{MAE}=\frac{1}{m}\sum_{i=1}^{m}\left|y_{i}-{\hat{y}}_{i}\right|$$

where $y_{i}$ is the real value and ${\hat{y}}_{i}$ is the predicted value.

### 6.2. Verification of the Clustering Effect

To verify the clustering effect and find the more suitable network for each cluster, we used LSTM and GRU to make predictions on different data. Firstly, we directly used LSTM and GRU networks to make predictions on the entire dataset that has not been clustered, and then we used LSTM and GRU to make predictions separately on the data of the four clusters we divided. The experimental results for the 2-s prediction and 5-s prediction are shown in Table 3 and Table 4, respectively. In the experiment, we randomly divide the training set and the test set multiple times to train the network. When using different training sets and test sets from the same distribution to train the network, the difference in RMSE of the results is small. Moreover, after modifying the learning rate, batch size, and other parameters, the results of multiple experiments also consistently fall within the same range. This indicates that our experimental results are highly reliable. The occurrence of random time is eliminated.

From the data in these two tables, it can be observed that, regardless of whether the same LSTM or GRU is used for acceleration prediction, half of the results are significantly improved after clustering. Furthermore, the average result, which takes the sample size into account, is also improved. This demonstrates that clustering data with similar driving characteristics before training prediction models can improve prediction accuracy.

In addition, by comparing the prediction results of LSTM and GRU on each cluster, it can be seen that the two networks have different performances. However, no network outperforms the other on all data clusters, which also underscores the importance of selecting the appropriate prediction network for each cluster. Based on the two tables above, we can select the most suitable prediction networks for each cluster according to their performance, which has been highlighted in red in the tables. LSTM networks based on Cluster 2, 3, 4, and 5 clustering data perform better. The performance of GRU networks based on Cluster 1 clustering data is superior.

**Figure 6:**
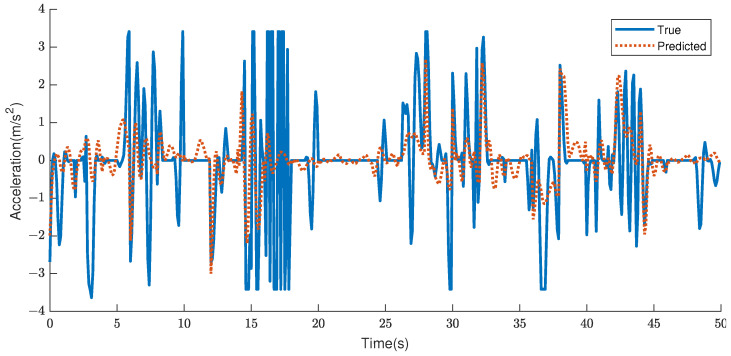
Sample simulated acceleration predicted by the LSTM network, compared to the true trajectories using the unclustered data. The graph depicts 25 individual 2-s trajectory for an individual ego vehicle.

**Figure 7:**
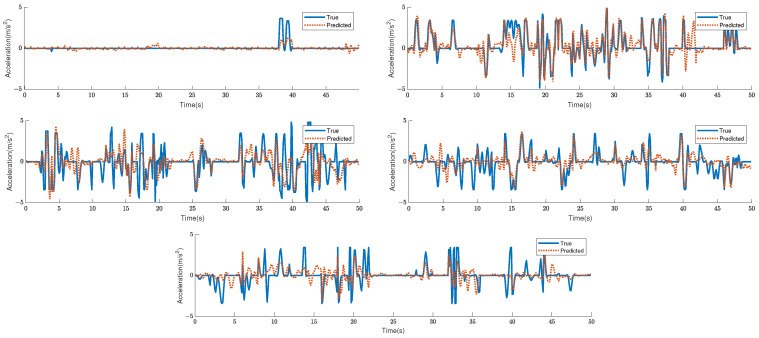
Sample simulated acceleration predicted by each better network, compared to the true trajectories after clustering. Each graph depicts 25 individual 2-s trajectory for an individual ego vehicle.

Based on the highlighted data, the prediction results for Cluster 1, 2, 4, and 5 are superior to those of unclustered data. Specifically, 46.7%, 7.5%, 4.2%, and 19.2% of the RMSEs are optimized for the 2-s prediction, whereas 40.8%, 6.9%, 2.3%, and 14.6% are optimized for the 5-s prediction. The RMSE of the averaged result is also optimized by 10.0% and 6.9% for 2-s and 5-s predictions, respectively. The projected acceleration at 2 s and the actual acceleration are depicted in Figure 6 and Figure 7, respectively.

It is worth noting that the results for Cluster 3 are even worse when compared to those of unclustered data. By looking at the data in Cluster 3, we found that the acceleration changes a lot and that Cluster 3 has the highest variation in acceleration. This makes it hard for LSTM and GRU to make good predictions with this type of data. Although these data also exist in the unclustered data and lead to poor prediction results, the better results of other clusters of data will average them out, making the results better than those of Cluster 3.

### 6.3. Comparison with Other Methods

Park and Ahn [11] used an enhanced DNN method for predicting acceleration by introducing an internal module for predicting driver intent prior to the acceleration prediction network and training the intent prediction network with human-defined inputs and desired outputs. The predicted future intentions of the driver and the underlying data are used as inputs for acceleration prediction. The final RMSE was calculated to be $1.977\;m/s^{2}$ using the same database as us.

Ref. [13] proposed an LSTM Neighbors network. They defined a set of “neighboring” vehicles surrounding the vehicle with 18 components of input state: vehicle acceleration, velocity, and the distance and velocity differences between the vehicle and the vehicles in front, behind, left, right, left front, left back, and right front, respectively. In addition to adopting the NGSIM database and using our model based on 2-s vehicle data to forecast 1-s and 5-s acceleration, the outcomes are displayed in Table 5. From the results, it can be seen that only the 5-s prediction result in Cluster 3 is slightly worse than theirs, while the remaining nine results are not worse than theirs.

## 7. Conclusions

In this paper, a clustering-prediction model for predicting vehicle acceleration is proposed. The main innovation is to divide the acceleration processes into different clusters with different acceleration patterns, and then choose different deep learning prediction networks for each cluster, thereby improving the adaptability and fitting of both the prediction network itself and the hyperparameters, and achieving the goal of improving acceleration prediction accuracy. To distinguish between different acceleration patterns, GMM is used to cluster the vehicle driving data segment. GMM is used to cluster the vehicle driving data segment in order to distinguish between different acceleration patterns. During clustering, regularization is used to prevent the model from overfitting. Each cluster in the clustered data has its own acceleration characteristics, and therefore, may require a different prediction network. LSTM and GRU networks are tested for the data in each cluster, and the better-performing network is then selected for each cluster. The experimental results demonstrate that the proposed method performs well in terms of improving acceleration prediction accuracy.

The method proposed in this paper can be used in vehicle fuel control, where fuel allocation is made more rational by predicting the driver’s behavior in order to reduce energy consumption and keep the vehicle running longer. It can also be used in vehicle safety systems, where the driver’s behavior is predicted in order to make advanced predictions and countermeasures for potential hazards.

## Figures and Tables

**Table 1:** AIC and BIC values of different clustering schemes.

Number of Cluster	2	3	4	5	6
AIC	495,020	479,090	376,340	360,820	371,040
BIC	496,130	480,770	378,590	363,630	374,410

**Table 2:** Parameters for experimental LSTM and GRU networks.

Environment	Number of Neurons	Loss Function	Optimizer	Learning Rate
PyTorch	128	MSE	Adam	0.001
Activision	Epoch	Batch	Dropout	Bidirectional
Function	Rate	Size		
Tanh	100	10	0.3	TRUE

**Table 3:** Results for 2-s prediction. The red part represents the better result.

	Sample	Prediction	RMSE	MAE
	**Size**	**Network**	**m/s${}^{2}$**	**m/s${}^{2}$**
Cluster 1	29,200	LSTM GRU	0.68 0.64	0.28 0.23
Cluster 2	879,800	LSTM GRU	1.11 1.24	0.71 0.83
Cluster 3	1,142,400	LSTM GRU	1.33 1.54	0.87 1.04
Cluster 4	2,348,600	LSTM GRU	1.15 1.27	0.79 0.84
Cluster 5	2,842,400	LSTM GRU	0.97 1.12	0.59 0.67
Average of Clusters	-	LSTM GRU	1.09 1.23	0.70 0.78
Average of the best combination	-	-	1.08	0.69
Entire dataset	7,505,200	LSTM GRU	1.20 1.32	0.78 0.83

**Table 4:** Results for 5-s prediction. The red part represents the better result.

	Sample	Prediction	RMSE	MAE
	**Size**	**Network**	**m/s${}^{2}$**	**m/s${}^{2}$**
Cluster 1	29,200	LSTM GRU	0.82 0.77	0.36 0.34
Cluster 2	879,800	LSTM GRU	1.21 1.33	0.81 0.90
Cluster 3	1,142,400	LSTM GRU	1.44 1.57	0.98 1.08
Cluster 4	2,348,600	LSTM GRU	1.27 1.34	0.89 0.90
Cluster 5	2,842,400	LSTM GRU	1.11 1.18	0.69 0.70
Average of Clusters	-	LSTM GRU	1.21 1.29	0.81 0.82
Average of the best combination	-	-	1.21	0.80
Entire dataset	7,505,200	LSTM GRU	1.30 1.35	0.84 0.84

**Table 5:** RMSE based on 2-s data over 1-s and 5-s horizons.

	1-s	Accuracy	5-s	Accuracy
		**Improvement**		**Improvement**
Jones	1.44	-	1.30	-
Cluster 1	0.69	52%	0.55	58%
Cluster 2	1.05	27%	1.28	2%
Cluster 3	1.22	15%	1.44	−11%
Cluster 4	1.03	28%	1.15	12%
Cluster 5	0.89	38%	1.12	14%

## Data Availability

Our original research dataset was sourced from data.transport.gov, the U.S. Department of Transportation’s public data portal. Link to show as follows: https://data.transportation.gov/Automobiles/Next-Generation-Simulation-NGSIM-Vehicle-Trajector/8ect-6jqj.

## Appendix A. Appendix Definitions of Driving Data Characteristics

The number of data points in a given driving segment is defined as *M*. The selected characteristics of driving data are defined as follows:

$$\begin{array}{c} \begin{array}{cc} & v_{av}=\frac{\sum_{i=1}^{M}v_{i}}{M}\\ & v_{sd}=\sqrt{\frac{1}{M}\sum_{i=1}^{M}{\left(v_{i}-v_{av}\right)}^{2}}\\ & v_{max}=max\left(v_{1},v_{2},\ldots ,v_{M}\right)\\ & v_{min}=min\left(v_{1},v_{2},\ldots ,v_{M}\right)\\ & a_{pav}=\frac{1}{p}\sum_{i}pa_{i}\\ & a_{nav}=\frac{1}{q}\sum_{i}qa_{i}\\ & a_{sd}=\sqrt{\frac{1}{M}\sum_{i=1}^{M}{\left(a_{i}-a_{av}\right)}^{2}}\\ & a_{max}=max\left(a_{1},a_{2},\ldots ,a_{M}\right)\\ & a_{min}=min\left(a_{1},a_{2},\ldots ,a_{M}\right)\\ & p_{a}=\frac{t_{a}}{M}\left(a>0.1\;{m/s}^{2}\right)\\ & p_{d}=\frac{t_{d}}{M}\left(a<-0.1\;{m/s}^{2}\right)\\ & p_{i}=\frac{t_{i}}{M}\left(-0.1<a<0.1\;{m/s}^{2}\right)\\ & s_{av}=\frac{\sum_{i=1}^{M}s_{i}}{M}\\ & s_{sd}=\sqrt{\frac{1}{M}\sum_{i=1}^{M}{\left(s_{i}-s_{av}\right)}^{2}}\\ & s_{max}=max\left(s_{1},s_{2}\ldots ,s_{M}\right)\\ & s_{min}=min\left(s_{1},s_{2},\ldots ,s_{M}\right) \end{array} \end{array}$$

## References

[1] Onori S., Serrao L., Rizzoni G. (2016). Hybrid Electric Vehicles: Energy Management Strategies.

[2] Chen Z., Liu Y., Zhang Y., Lei Z., Chen Z., Li G. (2022). A neural network-based ECMS for optimized energy management of plug-in hybrid electric vehicles. Energy.

[3] Cacciabue P.C. (2007). Modelling Driver Behaviour in Automotive Environments: Critical Issues in Driver Interactions with Intelligent Transport Systems.

[4] Carignano M.G., Costa-Castelló R., Roda V., Nigro N.M., Junco S., Feroldi D. (2017). Energy management strategy for fuel cell-supercapacitor hybrid vehicles based on prediction of energy demand. J. Power Sources.

[5] Ferdowsi A., Challita U., Saad W. (2019). Deep learning for reliable mobile edge analytics in intelligent transportation systems: An overview. IEEE Veh. Technol. Mag..

[6] Liang M., Yang B., Chen Y., Hu R., Urtasun R. Multi-task multi-sensor fusion for 3d object detection. Proceedings of the Proceedings of the IEEE/CVF Conference on Computer Vision and Pattern Recognition, Long Beach, CA, USA, 15–20 June 2019.

[7] Rezaei A., Burl J.B. (2015). Prediction of vehicle velocity for model predictive control. IFAC-PapersOnLine.

[8] Zou Y., Ding L., Zhang H., Zhu T., Wu L. (2022). Vehicle acceleration prediction based on machine learning models and driving behavior analysis. Appl. Sci..

[9] Liu K., Asher Z., Gong X., Huang M., Kolmanovsky I. (2019). Vehicle Velocity Prediction and Energy Management Strategy Part 1: Deterministic and Stochastic Vehicle Velocity Prediction Using Machine Learning.

[10] Montella A., Pariota L., Galante F., Imbriani L.L., Mauriello F. (2014). Prediction of drivers’ speed behavior on rural motorways based on an instrumented vehicle study. Transport. Res. Rec..

[11] Park S., Ahn C. (2019). Power management controller for a hybrid electric vehicle with predicted future acceleration. IEEE Trans. Veh. Technol..

[12] Du Y., Cui N., Li H., Nie H., Shi Y., Wang M., Li T. The vehicle’s velocity prediction methods based on rnn and lstm neural network. Proceedings of the 2020 Chinese Control Furthermore, Decision Conference (CCDC).

[13] Jones I., Han K. Probabilistic modeling of vehicle acceleration and state propagation with long short-term memory neural networks. Proceedings of the 2019 IEEE Intelligent Vehicles Symposium (IV).

[14] Hou J., Yao D., Wu F., Shen J., Chao X. (2021). Online vehicle velocity prediction using an adaptive radial basis function neural network. IEEE Trans. Veh. Technol..

[15] Fu R., Zhang Z., Li L. Using LSTM and GRU neural network methods for traffic flow prediction. Proceedings of the 2016 31st Youth Academic Annual Conference of Chinese Association of Automation (YAC).

[16] Wang Y., Wang C., Zhao W., Xu C. (2021). Decision-Making and Planning Method for Autonomous Vehicles Based on Motivation and Risk Assessment. IEEE Trans. Veh. Technol..

[17] Madhulatha T.S. (2012). An overview on clustering methods. arXiv.

[18] Kotsiantis S.B., Zaharakis I., Pintelas P. (2007). Supervised machine learning: A review of classification techniques. Emerg. Artif. Intell. Appl. Comput. Eng..

[19] Grira N., Crucianu M., Boujemaa N. (2004). Unsupervised and semi-supervised clustering: A brief survey. Rev. Mach. Learn. Tech. Process. Multimed. Content.

[20] (2016). Next Generation Simulation (NGSIM) Vehicle Trajectories and Supporting Data [Dataset]. Provided by ITS DataHub through Data.transportation.gov. Accessed Next Generation Simulation (NGSIM) Vehicle Trajectories and Supporting Data. https://datahub.transportation.gov/stories/s/Next-Generation-Simulation-NGSIM-Open-Data/i5zb-xe34/.

[21] Rabbath C., Corriveau D. (2019). A comparison of piecewise cubic Hermite interpolating polynomials, cubic splines and piecewise linear functions for the approximation of projectile aerodynamics. Defence Technol..

[22] Banfield J.D., Raftery A.E. (1993). Model-based Gaussian and non-Gaussian clustering. Biometrics.

[23] Zhao Y., Shrivastava A.K., Tsui K.L. (2018). Regularized Gaussian mixture model for high-dimensional clustering. IEEE Trans. Cybern..

[24] Vrieze S.I. (2012). Model selection and psychological theory: A discussion of the differences between the Akaike information criterion (AIC) and the Bayesian information criterion (BIC). Psychol. Methods.

[25] Jin X., Yu X., Wang X., Bai Y., Su T., Kong J. (2020). Prediction for Time Series with CNN and LSTM. Proceedings of the 11th International Conference on Modelling, Identification and Control (ICMIC2019).

[26] Ghodsi M., Liu X., Apfel J., Cabrera R., Weinstein E. RNN-transducer with stateless prediction network. Proceedings of the ICASSP 2020-2020 IEEE International Conference on Acoustics, Speech and Signal Processing (ICASSP).

[27] Yu Y., Si X., Hu C., Zhang J. (2019). A review of recurrent neural networks: LSTM cells and network architectures. Neural Comput..

[28] Gao S., Huang Y., Zhang S., Han J., Wang G., Zhang M., Lin Q. (2020). Short-term runoff prediction with GRU and LSTM networks without requiring time step optimization during sample generation. J. Hydrol..

[29] Yang S., Yu X., Zhou Y. LSTM and GRU Neural Network Performance Comparison Study: Taking Yelp Review Dataset as an Example. Proceedings of the 2020 International Workshop on Electronic Communication and Artificial Intelligence (IWECAI).

